# Effect of low level laser and low intensity pulsed ultrasound therapy on bone remodeling during orthodontic tooth movement in rats

**DOI:** 10.1186/s40510-018-0208-2

**Published:** 2018-04-16

**Authors:** Mohammed Mahmood Jawad Alazzawi, Adam Husein, Mohammad Khursheed Alam, Rozita Hassan, Rumaizi Shaari, Ahmad Azlina, M. S. Salzihan

**Affiliations:** 1 Orthodontics and Dentofacial Orthopedics, Eastman Institute for Oral Health, University of Rochester Medical Center, New York, USA; 2 School of Dental Sciences, Universiti Sains Malaysia, Health Campus, 16150 Kubang Kerian, Kelantan Malaysia; 3 Orthodontic Department, College of Dentistry, Al Jouf University, Sakaka, Saudi Arabia; 4 Orthodontic Unit, School of Dental Sciences, Universiti Sains Malaysia, Health Campus, Kubang Kerian, Kelantan Malaysia; 5 Clinical sciences, Faculty of Veterinary Medicine, Universiti Malaysia Kelantan, Kota Bharu, Kelantan Malaysia; 6 Biochemistry/Molecular biology, School of Dental Sciences, Universiti Sains Malaysia, Health Campus, Kubang Kerian, Malaysia; 7 Department of Pathology, School of Medical sciences, Universiti Sains Malaysia, Health Campus, Kubang Kerian, Kelantan Malaysia

**Keywords:** Orthodontics, Laser, Ultrasound, Bone remodeling, Tooth movement

## Abstract

**Background:**

Quality bone regeneration, which leads to the improvement of bone remodeling, is essential for orthodontic treatment. In order to improve bone regeneration and increase the amount of tooth movement, different techniques have been implemented. The object of this study is to compare the effects of low-level laser therapy (LLLT), low-intensity pulsed ultrasound (LIPUS), and their combination on bone remodeling during orthodontic tooth movement.

**Methods:**

Eighty (80) male, 6-week-old Sprague Dawley rats were grouped in to four groups, the first group was irradiated with (940 nm) diode laser, second group with LIPUS, and third group with combination of both LLLT and LIPUS. A forth group used was a control group in an incomplete block split-mouth design. The LLLT and LIPUS were used to treat the area around the moving tooth once a day on days 0–7, then the experiment was ended in each experimental endpoint (1, 3, 7, 14, and 21 days). For amount of tooth movement, models were imaged and analyzed. Histological examination was performed after staining with (hematoxylin and eosin) and (alizarin red and Alcian Blue) stain. One step reverse transcription-polymerase chain reaction RT-PCR was also performed to elucidate the gene expression of *RANK*, *RANKL*, *OPG*, and *RUNX-2*.

**Results:**

The amount of tooth movement, the histological bone remodeling, and the RT-PCR were significantly greater in the treatment groups than that in the control group. Among the treatment groups, the combination group was the highest and the LIPUS group was the lowest.

**Conclusion:**

These findings suggest that LLLT and LIPUS can enhance the velocity of tooth movement and improve the quality of bone remodeling during orthodontic tooth movement.

## Background

Within orthodontic treatment, to obtain physiological tissue reactions around a tooth while avoiding the side effects of an external force, long-term therapy of as long as 2 to 3 years is required. Accordingly, faster tooth movement without harmful effects on periodontal tissue and alveolar bone has been an issue of significant interest to orthodontists as well as patients [1, 2].

Recently, the biostimulation effect of low-level laser treatment (LLLT) is used to reduce the discomfort and pain that is triggered by trauma or even by the forces applied from the orthodontic appliance on teeth [3, 4]. It is thought that this stimulation could also increase bone repair by promoting better bone tissue remodeling, which can be considered a way to accelerate and evolve orthodontic treatment [5, 6].

In vitro studies have shown effects of LLLT on cell cultures. The LLLT irradiations improve and simulate osteoclastic activity [7, 8]. Also, in vivo rat experiments, LLLT stimulated midpalatal suture bone regeneration during expansion [9] and increase the tooth movement [4, 5, 10], and LLLT irradiation improves connective tissues turnover with bone remodeling process acceleration by stimulating osteoblast and osteoclast cell proliferation and function, such as elevating the ALP and RANK/RANKL/OPG system during orthodontic tooth movement [6, 11–14]*.* Similarly, in vivo human studies have shown tooth movement acceleration, significant reduction in levels of pain during orthodontic treatment, and healthy response from periodontal tissues after LLLT irradiation [15].

Additionally, low-intensity pulsed ultrasound (LIPUS) is also considered as a non-invasive stimulation technique to improve bone healing. The ultrasound is an acoustic pressure wave with frequencies above that of human hearing limit; it is transmitted through and into biologic tissues and is being used widely in medicine as a non-invasive therapeutic, operative, and diagnostic tool [16]. LIPUS has a biologic effect in promoting tissue healing [17, 18]. LIPUS signal is of low intensity enough to be considered neither thermal nor destructive [19]*.*

During in vitro studies, LIPUS stimulation affected osteogenic cells, leading to mineralized nodule formation. LIPUS have an essential effect on key functional activities of osteoblasts in bone [19, 20]. In in vivo rat studies, LIPUS improved bone fracture healing [21]. LIPUS accelerated bone regeneration of non-critical rat calvarial defects. Also, LIPUS accelerated osteoporotic fracture healing by enhancing callus remodeling, angiogenesis, and callus formation [20, 22]. Other studies compared the LLLT and LIPUS to stimulate bone fractures healing. By analyzing the effects of LIPUS and LLLT on the bone healing process, both devices LIPUS and LLLT could accelerate the bone healing process of fractured bones and after osteotomy in rats [23, 24].

The success of LIPUS to reduce bone fracture healing time may make it a promising tool for improving orthodontic treatment. The studies showed that LIPUS enhances mandibular growth in growing animals and humans. Additionally, LIPUS reduces the root resorption in humans. Also, LIPUS stimulates a significant increase in cementum and predentin formation and an increase in sub-odontoblast and periodontal ligament cell numbers [25–29].

Also, using both LLLT and LIPUS combined may increase the beneficial stimulatory effect compared with using each one of them alone. The rationality behind it is that the LLLT by its electromagnetic waves will stimulate the cells mitochondria and energy cell cycle, while at the same time, the LIPUS physical vibration will stimulate a natural functional movement around the cell membrane. By using them together, there is a possibility of getting a higher stimulatory response. However, the comparison between LLLT and LIPUS is still not well investigated for orthodontic treatment stimulation. Thus, the aim of this research is to study in vivo the effect of LLLT, LIPUS, and the combination of both techniques on orthodontic tooth movement.

## Methods

### Animals housing

The animal experimental protocol in this study was approved by the Ethics Committee for Animal Experiments in USM with the number USM/Animal Ethics Approval/2012/(77) (396). The sample size of rats was calculated by PS software version 3.0.10. The experiment used 80 Sprague Dawley rats (ARASC, USM, Malaysia), 6-week-old, weighing 180 ± 10 g. They were kept in the animal house of USM in separate cages in a 12-h light/dark environment at a constant temperature of 23 °C and provided with nutrients. The health status of each rat was evaluated daily and body weight monitored biweekly starting 1 week before the experiments; the body weight was not allowed to drop more than 15% during the experiment.

### Study design

The rats were grouped into three main experimental groups, the LLLT, LIPUS, and combination group. Additionally, two kinds of controls were used, an incomplete block split-mouth design as an “orthodontic treatment positive control” and “orthodontic treatment negative control” without any orthodontic treatment or any intervention. The experiment was terminated in days 1, 3, 7, 14, and 21. Each experimental and control group consisted of five rats for each endpoint, with a total of 25 rats for the experimental group.

### Experimental tooth movement

**Fig. 1:**
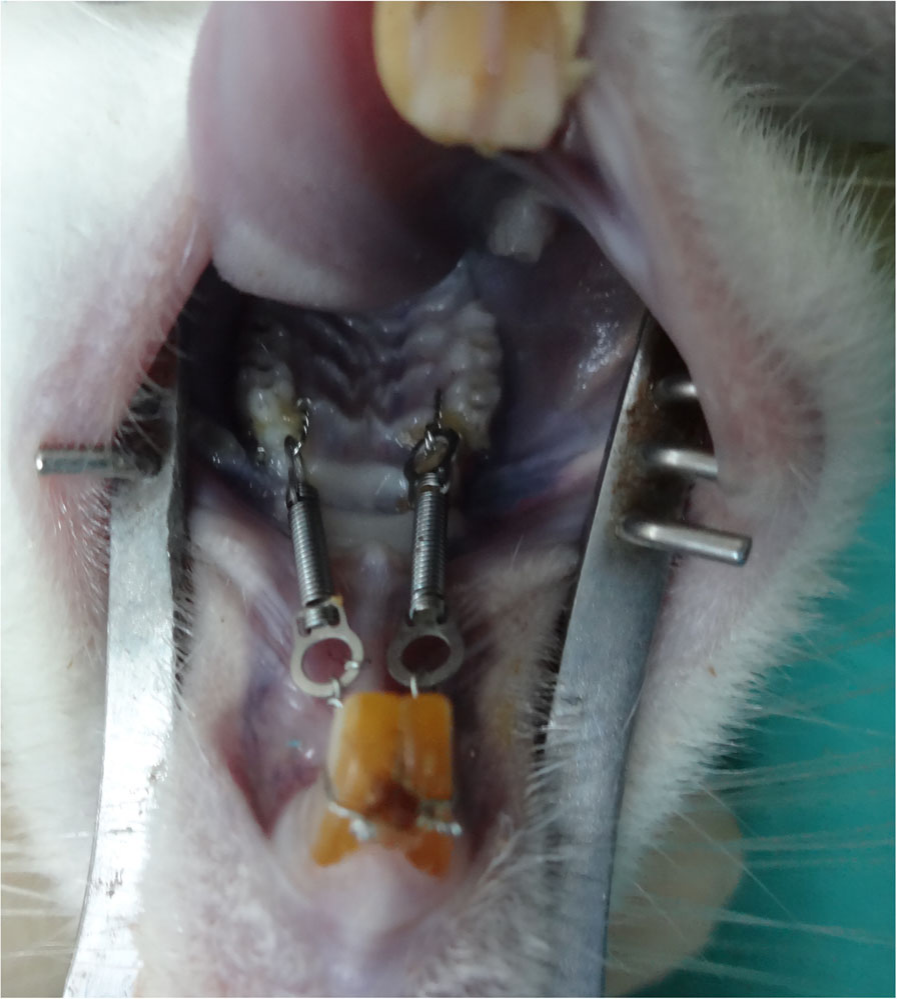
Orthodontic appliance in rat after placement

The animals were anesthetized with an intramuscular injection of ketamine hydrochloride (PUTNEY, USA) and xylazine hydrochloride (PROXYLAZ, Belgium) with 1 mg/kg body weight prior to any procedure involving them. Experimental tooth movement was performed according to the method of Fujita et al. [5] and Yamaguchi et al. [4] with a closed-coil spring (wire size 0.005 in., diameter 1/12 in.) (3 M Unitek, USA) ligated to the maxillary first molar by a 0.008 in. ligature wire (3 M Unitek, USA) [8, 9]. The other side of the coil spring was also ligated to the maxillary incisors using the same ligature wire. The upper first molar was moved mesially by the closing-coil spring with a force of 10 g that was measured by Dontrix gauge (OSE, USA). Figure 1 shows the orthodontic appliance.

### Laser irradiation

For the LLLT and combination groups, the laser device used was Ga-Al-As diode laser (ezlase, USA) with a wavelength at 940 nm in a continuous mode of operation. The laser power was 100 mW for 6 min/day with energy densities of 45.85 J∕cm^2^. The working powers and time of exposure were from Jawad et al. [11]. The laser beam was delivered by placing the end of the optical fiber tip in contact with the mesial, buccal, and palatal sides of the gingiva, located in the area of orthodontic movement as recommended by Fujita et al. [5] and Yamaguchi et al. [4]. Irradiation was performed once a day on days 0–7; then the experiment was ended in each experimental end point.

### LIPUS treatment

The rats for the LIPUS group and for the combination group were subjected to LIPUS generating 1.5 MHz frequency pulses, with a pulse width of 200 μs, repeated at 1 kHz, at an intensity of 30 mW/cm^2^ was used (Exogen, Smith & Nephew, USA). The end of the LIPUS transducer tip was placed in contact with the buccal side of the gingiva, located in the area of orthodontic movement. LIPUS stimulation was performed for 20 min daily for the days 0–7 as recommended by El-Bialy et al. [29].

### Measurement of tooth movement

**Fig. 2:**
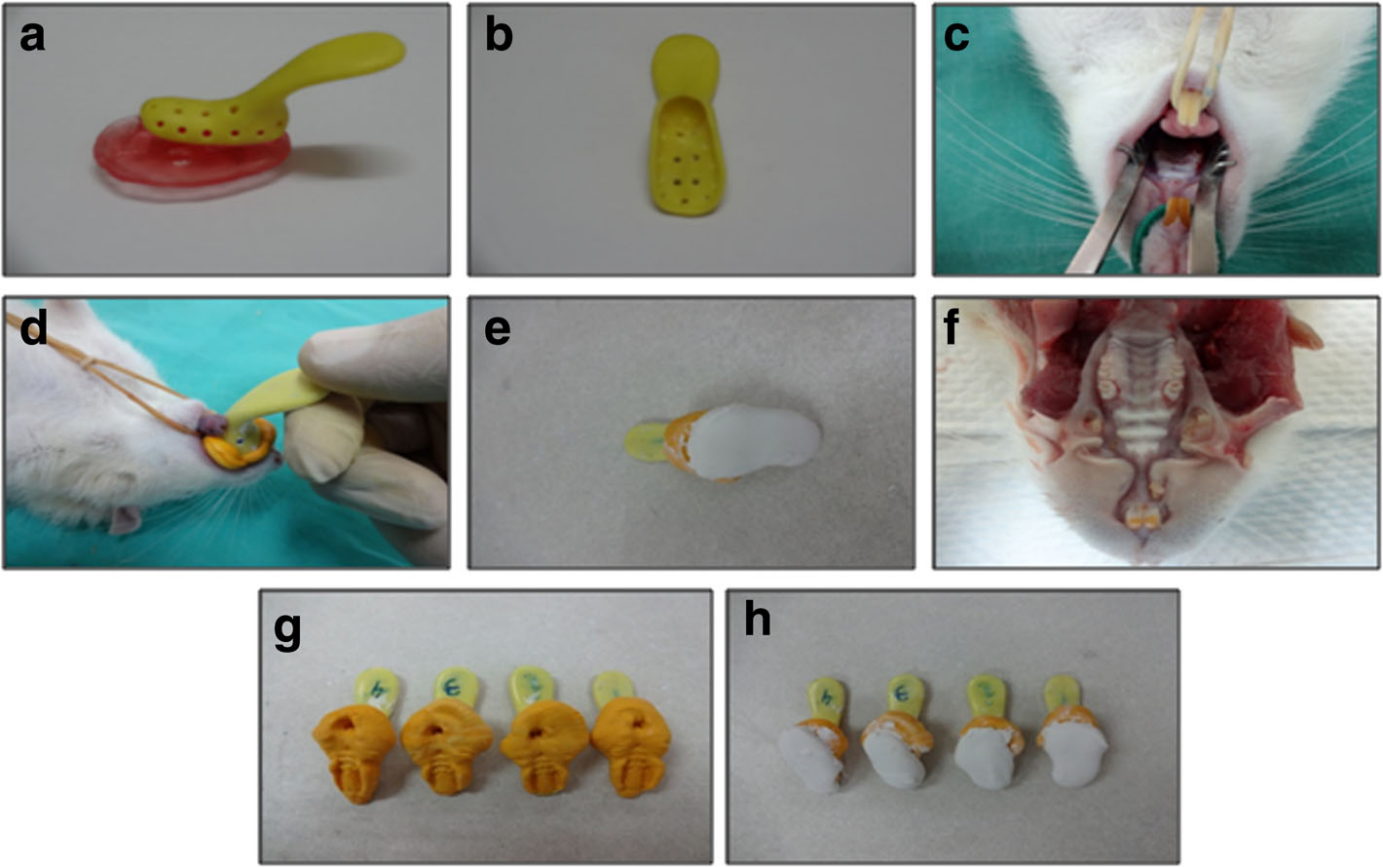
Rats impressions and study models formation. **a** fabrication of impression tries, **b** impression tray ready to be used, **c** rat in the position for taking impression, **d** taking first impression, **e** making first study model, **f** rat maxilla after animal sacrificed and orthodontic appliance was removed, **g** taking second impression, **h** making second study model

To determine the amount of tooth movement, impressions for rat’s maxilla were taken before orthodontic appliances placement and in the end of the experimental time point. Then, study models were made. The models were used to determine the amount of tooth movement by comparing before and after study models for all animals of the experiment. The models’ image was taken by medical image analysis system (JVC, USA), using Leica Material Workstation analysis software version 3.2.1. Measurement was made to the distance between the first molar central fossa and the second molar mesial surface to determine tooth movement in animals of the experiment as done by Yamaguchi et al. [4]. Figure 2 shows the study models formation.

### Tissue preparation

**Fig. 3:**
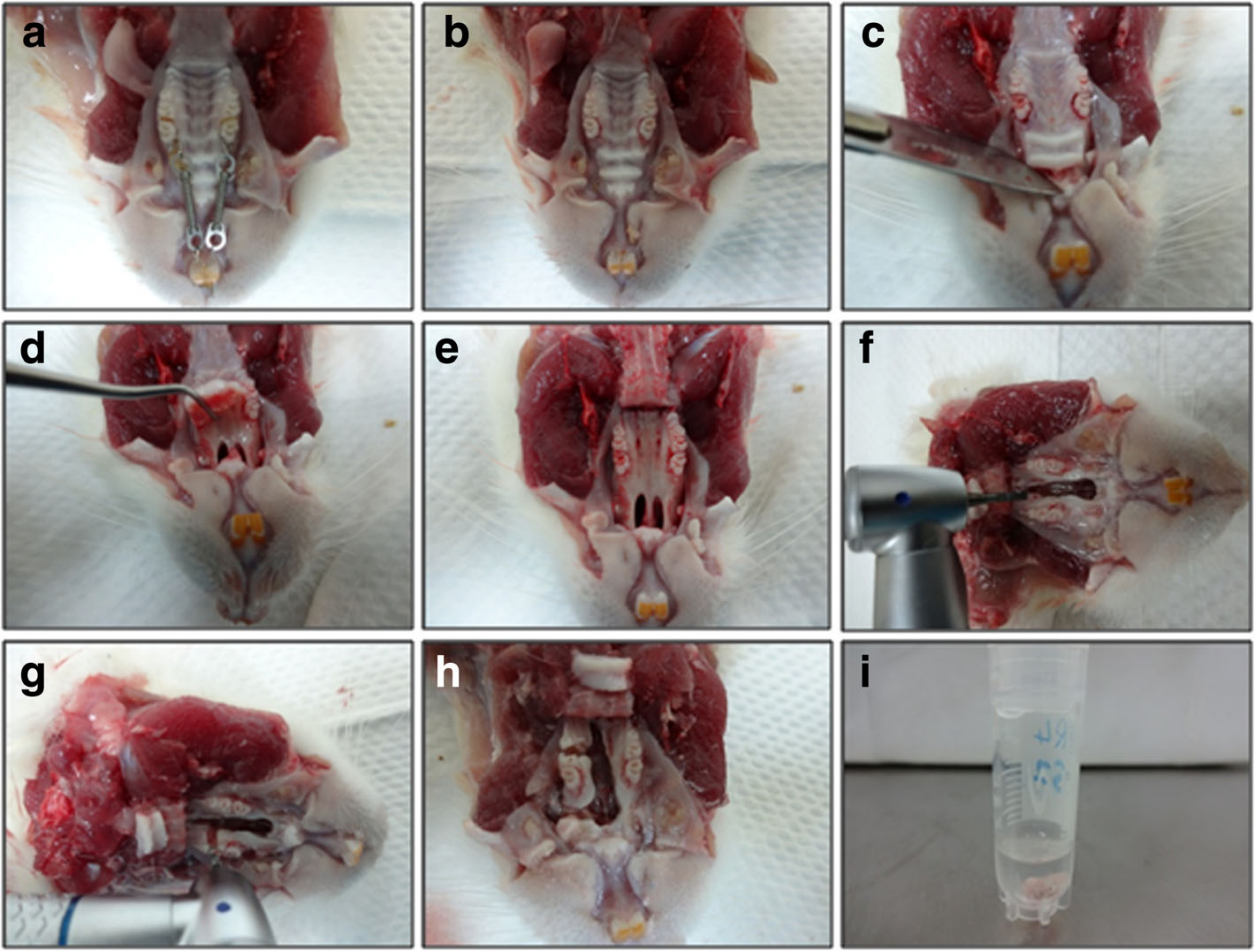
Procedure of tissue dissection. **a** Maxilla detached from mandible. **b** Orthodontic appliance removed. **c** Cutting gingiva. **d**, **e** Gingival detachment. **f**, **g**, **h** Cutting the area of interest. **i** Preserving tissue specimens

At the end of each experimental period, rats were terminated by using 100% CO_2_ inhalation as recommended be ARASC. After that the maxilla was immediately dissected using surgical instruments and low-speed handpiece. The cut on the tissue was 2 mm mesial and distal to the first molar which was the area of interest that contains the alveolar bone with the resorption and deposition sites and the PDL. Then, the tissue specimens were immersed in preservative solution as shown in Fig. 3.

### RNA isolation and RT-PCR

The tissue specimens for the experimental period were subjected to one step reverse transcription-polymerase chain reaction (RT-PCR). TRIzol reagent kit (Ambion, USA) was used to dissolve the tissue samples in order to extract the RNA. The NCBI BLAST server was used to determine primer specificity for the expression of the following genes: NF-_*K*_B ligands (*RANKL*), NF-_*K*_B (*RANK*), Osteoprotegerin (*OPG*), Runt-related transcription factor 2 (*RUNX2*), and Glyceraldehyde 3-phosphate dehydrogenase (*GAPDH*). The primer sequences, melting temperature, product size, and gene bank accession number are shown in Table 1. Each RNA expression level was measured as the ratio of each gene relative to the *GAPDH* expression level using semi quantitative analysis. RT-PCR was performed following the QIAGEN OneStep RT-PCR kit (QIAGEN, Germany) manufacturer’s protocol.

**Table 1:** The primer sequences, melting temperature, their respective product size, and gene bank accession number

Target Gene	Primers	Melting T_m_ (°C)	Product size	Gene bank accession no.
RANKL	Forward:5′-acgcagatttgcaggactcgac-3′	59.5	493 bp	AF019048
	Reverse:5′-ttcgtgctccctcctttcatc-3′	57.6		
RANK	Forward:5′-ttaagccagtgcttcacggg-3′	57.4	497 bp	AF018253
	Reverse:5′-acgtagaccacgatgatgtcgc-3′	59.5		
OPG	Forward:5′-tggcacacgagtgatgaatgcg-3′	59.5	537 bp	U94330
	Reverse:5′-gctggaaagtttgctcttgcg-3′	57.6		
RUNX2	Forward:5′-gaaccaagaaggcacagaca-3′	55.4	452 bp	NM053470.2
	Reverse:5′-tccaccaccctgttgctgta-3′	55.4		
GAPDH	Forward:5′-accacagtccatgccatcac-3′	57.4	452 bp	NM017008
	Reverse:5′-tccaccaccctgttgctgta-3′	57.4		

The PCR was performed with a Mastercycler pro thermal cycler. PCR was conducted for 30 cycles of denaturing at 94 °C for 45 S, annealing at 60 °C for 1 min, and extension at 72 °C for 1 min. PCR for *GAPDH* used as an endogenous control was carried out in parallel under the same conditions. The PCR products were analyzed using agarose gel. Agarose gel of 2% was made by mixing 0.5 g of agarose powder with 25 ml of 1× LB buffer in a glass beaker with 2.5 μl of SYPR Safe DNA gel stain then heated the mixture in a microwave for 1 min until the agarose powder had completely dissolved. A gel casting comb was inserted in its gel casting tray. After that, the gel was poured into gel cast and left to hard for 30 min in a dark place. When the gel became hard, it was immersed in electrophoresis tank containing 1× LB buffer. The PCR product of 5 μl was mixed with loading dye of 1 μl and was loaded in the well of agarose gel. One hundred base pair DNA ladder of 1 μl mixed with loading dye of 1 μl was usually loaded in the first left well in agarose gel. To run the gel, the gel was subjected to electrophoresis for 60 min at 70 V and visualized under ultraviolet light of Gel doc electrophoresis image analyzer system using Quality One software (Bio-Rad, V 4.6.7). Each RNA expression level was measured as the ratio of each gene relative to the *GAPDH* expression level using semi quantitative analysis.

### Tissue processing, hematoxylin/Eosin, and Alizarin red /Alcian Blue staining

The specimens were decalcified in 10% disodium ethylenediamine tetra-acetic acid (pH 7.4) solution for 4 weeks. Then, the specimens were casted by paraffin wax. The microtome was used to slice sample into 4 μm continuous sections in the horizontal direction and mounted on glass slides to be used for further staining.

For staining, the slides were rinsed in distilled water and stained in hematoxylin (Merck, Germany). Slides were counterstained in eosin (Sigma-Aldrich, USA) and dehydrated, cleared, and mounted. Slides were viewed with slide scanner (Zeiss, Germany).

Also, alizarin red was used to identify calcium in tissue sections such as the bone, and the Alcian Blue was used to identify mucosubstances of connective tissues. The first step for staining is deparaffinization then rehydration and staining with Alcian Blue solution (Sigma-Aldrich, USA). Slides were washed and stained in alizarin red stain solution (Sigma-Aldrich, USA). Slides were dehydrated, cleared, and mounted. Slides were viewed with slide scanner (Zeiss, Germany).

## Statistical analysis

The statistical analyses of the data for all experiments were performed using Statistical Package of Social Science (SPSS) software version 22 (Armonk, NY: IBM Corp. Released 2013). For teeth movement measurement and gene expression analysis, the assumptions of normality and homogeneity of variances were checked and were not fulfilled; Kruskal-Wallis Test was used. The pairwise comparisons were analyzed through Mann-Whitney test and Bonferroni correction. A value of *P* < 0.05 was considered statistically significant.

## Results

### Measurement of tooth movement

**Fig. 4:**
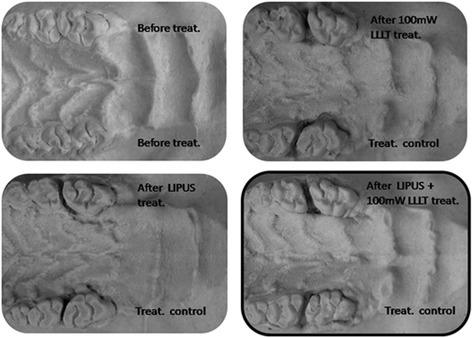
Study model images showing the effect of LLLT, LIPUS, and combination of them on orthodontic tooth movement in rats at day 21

**Fig. 5:**
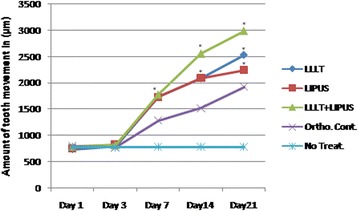
Effect of LLLT, LIPUS, and combination of them on orthodontic tooth movement in rats at days 1, 3, 7, 14, and 21. At days 14 and 21, all the treatment groups were significantly higher than the control group. Among the treatment groups, the combination group was the highest, while the LIPUS group was the lowest; the data are shown as the mean ± SEM of three separate experiments. *P ≤ 0.05 between groups

When comparing the amount of tooth movement among the experimental groups at days 1 and 3, the results showed that there were no significant differences among them. The comparison at day 7 showed that all the treatment groups were significantly higher than the control group, but there was no significant difference between the treatment groups. The comparison at day 14 showed that all the treatment groups were significantly higher than the control group. Among treatment groups, the combination group was the highest, while there was no significant difference between the LLLT and LIPUS groups. The comparison at day 21 showed that all the treatment groups were significantly higher than the control group. Among the treatment groups, the combination group was the highest, while the LIPUS group was the lowest, as shown in Table 2, Figs. 4 and 5.

**Table 2:** Comparison of median for the tooth movement measurements among study groups at day 21

Comparison of groups	*n*	Median (IqR)	X^2^-Statistic^a^ (df)	*P* value^b^
Control	5	1923.736 (1.93)		
LLLT 100 mW	5	2531.897 (3.64)		
LIPUS	5	2249.468 (6.20)	17.857 (3)	0.000
LLLT 100 mW + LIPUS	5	2990.864 (3.85)		

*IqR* interquartile range

^a^Kruskal-Wallis test was applied

^b^Mann-Whitney test with Bonferroni correction for individual pair was applied, *P* < 0.001 between all pairs

### Gene expression analysis

**Fig. 6:**
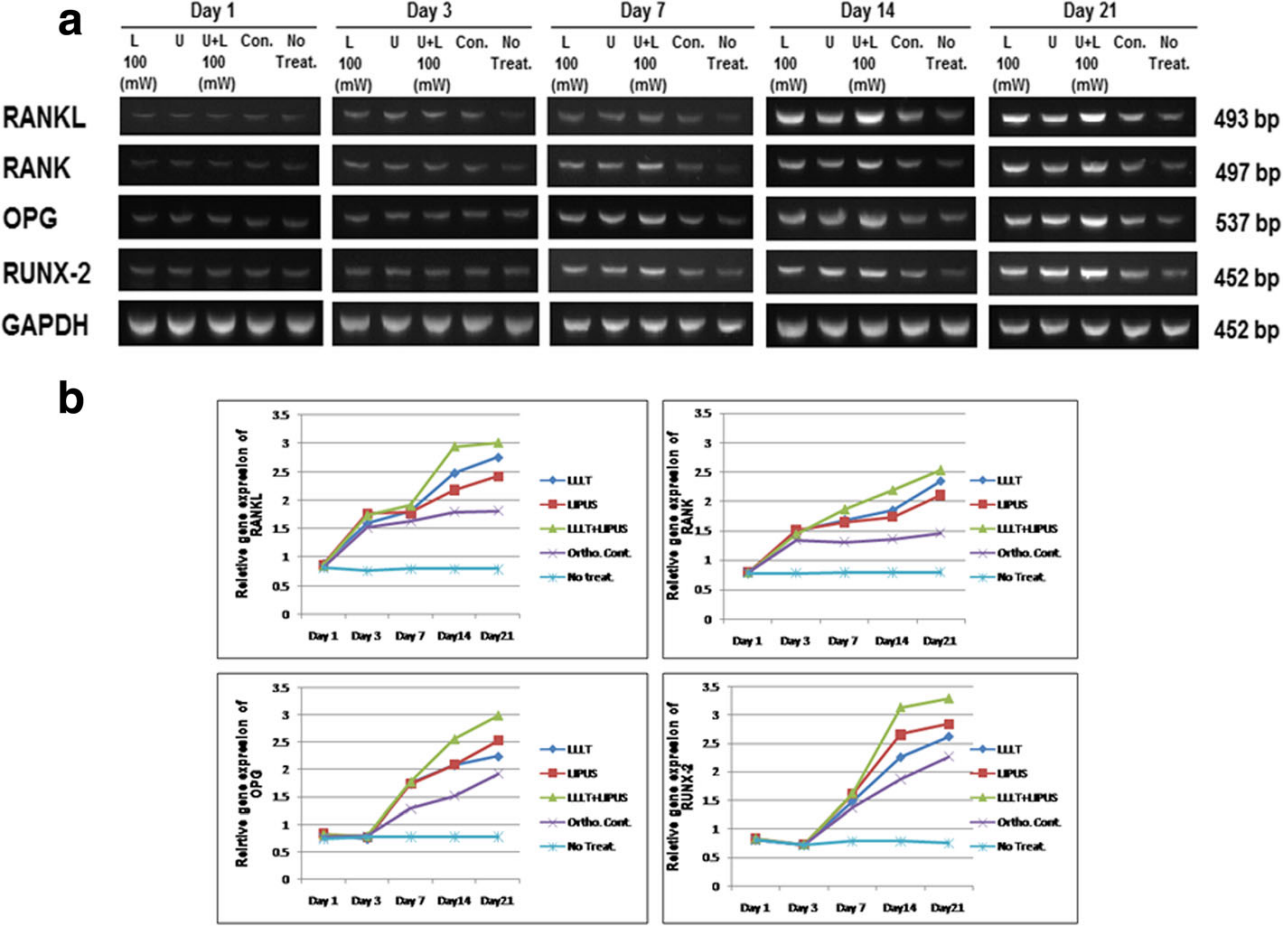
Effect of LLLT, LIPUS, and combination on gene expression of rat’s maxilla at days 1, 3, 7, 14, and 21. The gene expression for *RANKL*, *RANK*, *OPG*, and *RUNX2* was determined by using semi-quantitative RT-PCR and normalized by *GAPDH* for the following groups: non- treatment, orthodontic control, LLLT (100 mW), LIPUS, and (LLLT + LIPUS). All genes were significantly upregulated in the treatment groups in relation to the orthodontic controls. Between the treatment groups, *RANKL* and *RANK* were the highest in the combination group followed by the LLLT group. For *OPG* and *RUNX2*, the combination group was the highest and followed by the LIPUS group. The data are shown as the mean ± SEM of three separate experiments. *P ≤ 0.05 between groups

At day 1, there was no upregulation in any experiment gene with any of the groups. At day 3, *RANKL* and *RANK* upregulated in treatment and orthodontic control groups in relation to none treated group, but there was no significant difference between the treatment groups and orthodontic control or between the treatment groups themselves. For *OPG* and *RUNX2*, there was no upregulation in any groups. At day 7, *RANKL* and *RANK* upregulated in the treatment groups in relation to orthodontic control. Between the treatment groups, the combination group was the highest. For *OPG* and *RUNX2*, there was upregulation in the treatment groups in relation to orthodontic control. Between the treatment groups, there were no significant differences. At days 14 and 21, all genes were upregulated in the treatment groups in relation to orthodontic control. Between the treatment groups, *RANKL* and *RANK* were the highest in the combination group followed by the LLLT group. For *OPG* and *RUNX2* also the combination group was the highest and followed by the LIPUS group as shown in Tables 3, 4, 5, and 6 and Fig. 6.

**Table 3:** Comparison of median of RANKL gene expression levels among the study groups at day 21

Comparison of groups	*n*	Median (IqR)	X^2^-Statistic^a^ (df)	*P* value^b^
Non-treat.	6	0.783 (0.19)		
Ortho. Control	6	1.806 (0.16)		
LLLT 100 mW	6	2.758 (0.17)	11.423 (4)	0.000
LIPUS	6	2.420 (0.16)		
LLLT 100 mW + LIPUS	6	3.014 (0.12)		

*IqR* interquartile range

^a^Kruskal-Wallis test was applied

^b^Mann-Whitney test with Bonferroni correction for individual pair was applied, *P* < 0.001

**Table 4:** Comparison of median of RANK gene expression levels among the study groups at day 21

Comparison of groups	*n*	Median (IqR)	*X*^2^-Statistic^a^ (df)	*P* value^b^
Non- treat.	6	0.789 (0.21)		
Ortho. Control	6	1.453 (0.19)		
LLLT 100 mW	6	2.346 (0.13)	22 .271 (4)	0.000
LIPUS	6	2.099 (0.16)		
LLLT 100 mW + LIPUS	6	2.528 (0.11)		

*IqR* interquartile range

^a^Kruskal-Wallis test was applied

^b^Mann-Whitney test with Bonferroni correction for individual pair was applied, *P* < 0.001

**Table 5:** Comparison of median of OPG gene expression levels among the study groups at day 21

Comparison of groups	*n*	Median (IqR)	*X*^2^-Statistic^a^ (df)	*P* value^b^
Non- treat.	6	0.782 (0.23)		
Ortho. Control	6	1.923 (0.21)		
LLLT 100 mW	6	2.248 (0.19)	10.783 (4)	0.000
LIPUS	6	2.531 (0.18)		
LLLT 100 mW + LIPUS	6	2.990 (0.22)		

*IqR* interquartile range

^a^Kruskal-Wallis test was applied

^b^Mann-Whitney test with Bonferroni correction for individual pair was applied, *P* < 0.001

**Table 6:** Comparison of median of RUNX2 gene expression levels among the study groups at day 21

Comparison of groups	*n*	Median (IqR)	*X*^2^-Statistic^a^ (df)	*P* value^b^
Non-treat.	6	0.752 (0.19)		
Ortho. Control	6	2.262 (0.21)		
LLLT 100 mW	6	2.622 (0.19)	8 .829 (4)	0.000
LIPUS	6	2.840 (0.21)		
LLLT 100 mW + LIPUS	6	3.285 (0.23)		

*IqR* interquartile range

^a^Kruskal-Wallis test was applied

^b^Mann-Whitney test with Bonferroni correction for individual pair was applied, *P* < 0.001

### Histological evaluation

**Fig. 7:**
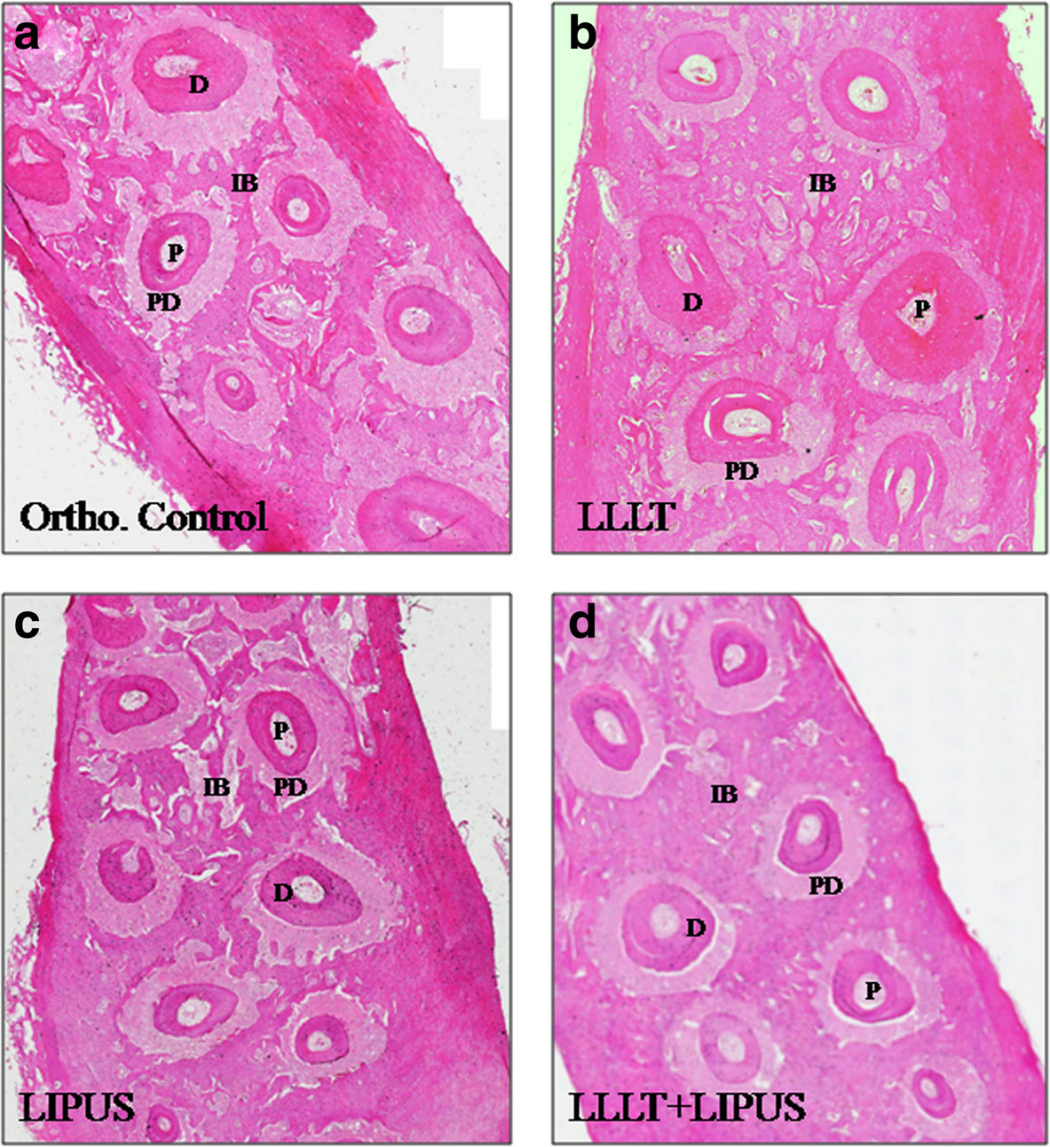
Light micrographs of rat’s maxilla portions with orthodontically induced tooth movement for 21 days. There was higher amount of interseptal bone between the roots of the teeth in the treatment groups in relation to the orthodontic control group which also showed wider periodontal ligaments. Between the treatment groups, the interseptal bone was highest in the combination group, followed by the LLLT group and then the LIPUS group, sections stained with H&E. × 10. **a** Orthodontic control, **b** treated with 100 mW LLLT, **c** treated with LIPUS, and **d** treated with 100 mW LLLT + LIPUS. *P*: pulp, *IB*: interseptal bone, *PDL*: periodontal ligament, *D*: dentin

**Fig. 8:**
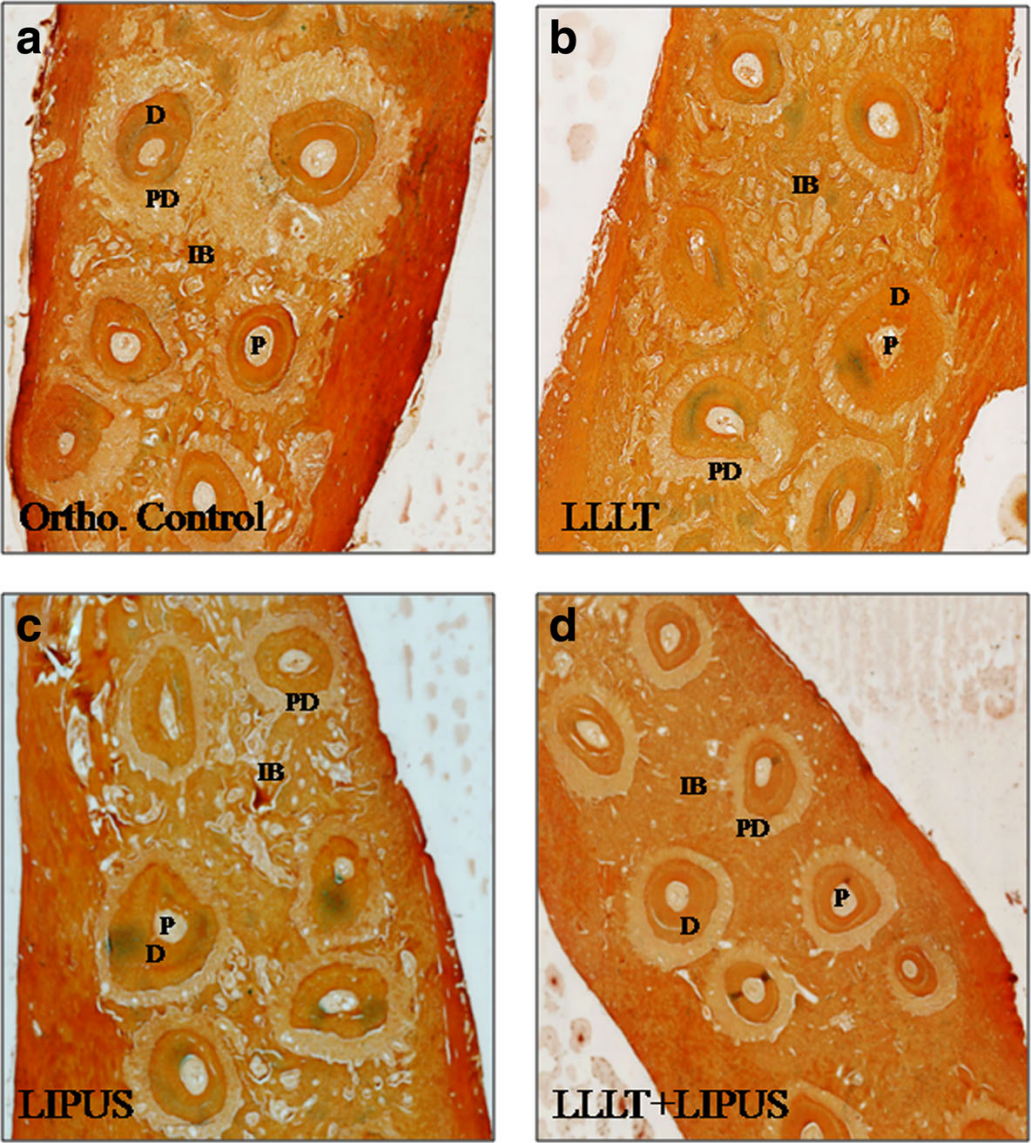
Light micrographs of rat’s maxilla portions with orthodontically induced tooth movement for 21 days. There was higher amount of interseptal bone between the roots of the teeth in the treatment groups in relation to the orthodontic control group which also showed wider periodontal ligaments. Between the treatment groups, the interseptal bone was highest in the combination group, followed by the LLLT group and then the LIPUS group, sections stained with alizarin red and Alcian Blue specialized staining. × 10. **a** Orthodontic control, **b** treated with 100 mW LLLT, **c** treated with LIPUS, and **d** treated with 100 mW LLLT + LIPUS. *P*: pulp, *IB*: interseptal bone, *PDL*: periodontal ligament, *D*: dentin

For histological evaluation, at days 1 and 3, there were no differences between all the experimental groups. At day 7, there was a higher amount of interseptal bone between the roots of the teeth in the treatment groups in relation to the orthodontic control group. Between the treatment groups, there was no difference. At day 14, there was higher amount of interseptal bone between the roots of the teeth in the treatment groups in relation to the orthodontic control group. Between the treatment groups, the interseptal bone was higher in the combination group, while there was no difference between the LLLT and the LIPUS groups. At day 21, there was higher amount of interseptal bone between the roots of the teeth in the treatment groups in relation to the orthodontic control group which also showed wider periodontal ligaments. Between the treatment groups, the interseptal bone was highest in the combination group, followed by the LLLT group and then the LIPUS group as shown in Figs. 7 and 8.

## Discussion

The purpose of this study was to compare the effects of LLLT, LIPUS, and combination of both LLLT and LIPUS on orthodontic tooth movement with the use of gene expressions and histological evaluation in order to determine the best stimulation method for in vivo in rats. For gene expressions, the *RANKL*, *RANK*, *OPG*, and *RUNX2* were used. The *OPG* and *RUNX2* gene expressions are considered as early markers for osteoblastic activity and bone formation [5]. On the other hand, *RANKL* and *RANK* are considered as early markers for osteoclastic activity and bone resorption [6]*.*

The results at day 1 can be attributed to the effect of orthodontic appliance and the LLLT, and the LIPUS stimulating methods were just starting and did not have enough time to show difference on tissue structure. Yamaguchi et al. [4] reported that at day 1, 100 mW of LLLT did not affect the orthodontic tooth movement as compared to non-irradiated area [4]. At day 3, the comparison of orthodontic tooth movement and histological evaluation showed that there was no significant difference among the study groups. This result is consistent with Altan et al. [6] who found that there was no statistical difference in tooth movement rate between the control group and 100 mW of LLLT irradiated group [6]. On the other hand, *RANKL* and *RANK* gene expressions were upregulated in the treatment and orthodontic control groups in relation to the none treated group. The upregulation of *RANKL* and *RANK* usually takes place when the osteoclasts are starting to mature and active. Starting bone resorption is the first step for tooth movement before the actual clinical tooth movement can be observed. It seems that the upregulation of *RANKL* and *RANK*, triggering the osteoclastic activity, was due to the orthodontic force that was applied by the orthodontic appliance and not due to LLLT or LIPUS, because this upregulation was in all the experimental groups of both treatment and orthodontic control groups with no significant difference among them. At day 7, the LLLT, LIPUS, and their combination groups showed better tooth movement with minimal intersptal damage or over resorption. This gives us an indication that LLLT and LIPUS stimulation effects started to appear.

At days 14 and 21, the results gave the indication that LLLT stimulates both osteoblastic and osteoclastic activity, but it is more effective towards osteoclastic activity stimulation. RANKL and RANK were the highest in the combination group followed by the LLLT group. For OPG and RUNX2 also the combination group was the highest followed by the LIPUS group as shown in Fig. 6. It seems that the combination effect on osteoclast pathway and on the osteoblast pathway is additive without impediment from the other pathways. For LIPUS, it is on the contrary, in spite of its stimulating effect for osteoclastic activity, it is more effective towards osteoblastic activity stimulation. For histological evaluation, higher amount of interseptal bone between the roots of the teeth was shown in the treatment groups in relation to the orthodontic control group. Among the treatment groups, the amount of interseptal bone was highest in the combination group followed by the LLLT group then by LIPUS group.

The results in general showed that the combination group had best stimulation effect on clinical tooth movement, bone gene expression levels, and histological bone formation that can be attributed to the synergistic effect of both LLLT and LIPUS during tooth movement in which the LLLT stimulates cell metabolic activities through stimulating cell mitochondrial energy cycle [30], and the LIPUS signals induce conformational changes in osteoblast cells membrane that alter ionic permeability and second messenger activity. The changes in the second messenger activity lead to downstream alterations in gene expression and resulting in an acceleration of the osteoblasts activity by upregulating bone-specific genes [31]. Using both LLLT and LIPUS combined increased the beneficial stimulatory effect compared with using each one of them alone. That is because the LLLT by its electromagnetic waves possibly stimulated the cell mitochondria and energy cell cycle, while at the same time the LIPUS physical vibration stimulated a natural functional movement around the cell membrane [32, 33]. To the best of our knowledge, there is a limited number of studies comparing the effect of LLLT, LIPUS, or their combination on orthodontic tooth movement, gene expression, or histological evaluation.

## Conclusion

The findings of this study suggest that the use of LLLT and LIPUS can increase orthodontic tooth movement, upregulate tissue gene expressions, and improve bone remodeling in the area of orthodontic tooth movement activity and especially when the two stimulating methods are combined together.

## Abbreviations

LLLT: Low-level laser therapy
LIPUS: Low intensity pulsed ultrasound
